# Academic and non-academic predictors of common mental health difficulties among university students during the COVID-19 pandemic

**DOI:** 10.3389/fpubh.2024.1441176

**Published:** 2024-09-17

**Authors:** Joanne Worsley, Amy Dryburgh, Jason C. McIntyre, Rhiannon Corcoran

**Affiliations:** ^1^Department of Primary Care and Mental Health, University of Liverpool, Liverpool, United Kingdom; ^2^Department of Psychology, University of Liverpool, Liverpool, United Kingdom; ^3^School of Psychology, Liverpool John Moores University, Liverpool, United Kingdom

**Keywords:** COVID-19 pandemic, student mental health, social isolation, academic stress, intolerance of uncertainty

## Abstract

**Introduction:**

Public concern for the mental health of university students has been rising over recent years. Newly arising stressors associated with the COVID-19 pandemic could contribute to further mental health burden for students. This study aimed to understand the mental health status of university students at an early stage in the pandemic and to identify academic, non-academic, and COVID-19-related predictors of common mental health difficulties at this time.

**Methods:**

This study examined how academic and non-academic predictors relate to common mental health difficulties using a cross-sectional sample of university students (*n* = 3817).

**Results:**

There were high levels of depression and anxiety during the pandemic, with more than 50% experiencing levels above the clinical cut offs. Academic stress, social isolation, intolerance of uncertainty, and more negative attitudes towards remote teaching and learning predicted higher levels of depression and anxiety. University identification predicted lower levels of depression whereas receiving a diagnosis of COVID-19 was associated with higher levels of depression.

**Discussion:**

This study identified COVID-19-related factors that uniquely contributed to students’ distress during the pandemic, over and above social connectivity variables. As COVID-19 factors, such as the uncertainty surrounding the pandemic, may have driven an increase in distress levels among students, these findings provide insights that could help universities and policymakers develop targeted interventions to support the mental health and well-being of university students during future crises.

## 1 Introduction

The COVID-19 pandemic has posed risks to public mental health worldwide. Although public concern for the mental health of university students has been rising over recent years, the COVID-19 pandemic posed further challenges, including the suspension of in-person teaching as well as restrictions on traveling which forced many students to leave their term-time residences (1). Early evidence assessing the mental health implications of COVID-19 has identified a heightened prevalence of distress. For example, Chen and Lucock (2) reported high levels of depression and anxiety in a UK student sample, with more than 50% experiencing levels above the clinical cut offs. Longitudinal studies in Italy, Switzerland, and the UK reported poorer mental health and wellbeing outcomes following the onset of the COVID-19 pandemic (3–5).

Academic programs are demanding due to challenging curricula, rigorous workloads, as well as both intellectual and emotional demands. As a result, researchers have applied frameworks such as the Job Demands-Resources model (JD-R) to academic contexts [e.g., (6, 7)]. In the JD-R framework, demands lead to strain whereas resources are those aspects of the environment that reduce demands and help individuals to achieve work goals (8). Using the JD-R model, Pluut and colleagues examined the impact of stressors and resources on student wellbeing and academic performance and found that academic stressors contributed to low wellbeing among university students (7). Consistent with the JD-R model, McIntyre and colleagues found that university students are subjected to a range of stressors that contribute to distress (6). Assessment stress consistently emerged as the strongest academic predictor of poor mental health (6). The change to remote learning and loss of daily student life routines potentially intensified pre-existing academic stress, contributing to further mental health burden for students (9).

In a recent longitudinal study exploring psychosocial and lifestyle variables, university connectedness was found to be the most notable predictor of internalizing symptom trajectories in a sample of first-year undergraduate students (10). Aspects of university connectedness encompasses one’s relationship with their peers, as well as involvement in group activities, events, and sports on campus. When examining both academic and non-academic predictors of student psychological distress, loneliness was identified as the strongest predictor of depression and anxiety while university friends were the most important social group with whom to identify in order to protect against depression and anxiety (6). In accordance with the Social Cure Model of health (11), a group has the potential to enhance health outcomes when individuals feel as though they bond with the members in the group and the group becomes incorporated into their sense of self through the process of social identification (12). Consistent with this model, identifying with more groups has been associated with reduced depression and anxiety among university students during stressful periods (13). Once an individual identifies with a group, they are more likely to receive and benefit from social support provided by this group (14). Taken together, this evidence suggests that enabling students to form a sense of identification with their peers and their institution may represent a psychological resource that improves mental health. However, as the COVID-19 pandemic has resulted in substantial reductions in recreational and leisure opportunities for students (10), the pandemic-associated restrictions are likely to have made it harder for students to identify with the university and their peers (15).

Perhaps the most obvious psychological mechanism associated with the COVID-19 pandemic is the uncertainty that defines the extent and timescale of the lifestyle restrictions. In general, high intolerance of uncertainty is associated with higher levels of mental distress, particularly anxiety (16). The intolerance of uncertainty model suggests that individuals whose tolerance for uncertainty is low have the tendency to respond negatively to situations that are uncertain (17). The COVID-19 pandemic is, by definition, an uncertain event of global significance that has no clear end date or outcome for individuals or societies. When combined with multiple lockdowns, uncertainty intolerance has been found to increase mental distress (18). Indeed, students have faced a number of further uncertainties during this time, including inadequate information regarding exams and graduation, losing their part-time jobs, and difficulties in managing their new study life (19). This suggests that the increase in mental distress reported by students may be linked to the extent to which they tend to tolerate uncertain situations and circumstances.

We aimed to understand the mental health of university students during the COVID-19 pandemic and identify academic and non-academic predictors of student psychological distress. In line with our previous findings [see (6)], we expected that (1) higher levels of academic stress and expectations stress, (2) low sense of identification with university and university friends, and (3) higher levels of social isolation would be associated with higher levels of depression and anxiety. Further, in the context of the pandemic, we expected that (4) intolerance of uncertainty, (5) having contracted COVID-19, and (6) dissatisfaction with online teaching would be associated with higher levels of depression and anxiety.

## 2 Methods

### 2.1 Ethical approval

Ethical approval was received from the Institute of Population Health Sciences (IPHS) Research Ethics Committee. All participants have given consent for their data to be used in the research.

### 2.2 Design

The cross-sectional online survey was conducted during the latter part of October 2020 and the first part of November 2020.

### 2.3 Procedure

An online survey link was sent via email to all students registered at one of two large universities in the North of England. The online survey was open to all students of these institutions. The survey was designed to be completed within approximately 20 min. To ensure broad participation, a reminder email was sent out 1 week after the initial invitation. Participation was voluntary, and students were informed that their responses would be anonymous and confidential. The survey was accessible for a period of 2 weeks, during which students could complete it at their convenience. The data collection was conducted in the period between 29.10.2020 and 12.11.2020. To put this in context, England entered the first national lockdown in March 2020. Following partial lockdown lifting in Summer 2020, restrictions were reintroduced in England during September 2020 (e.g., indoor and outdoor gatherings of six or more people were banned and there was a return to working from home). The second national lockdown in England was announced (on 31st October 2020) and implemented (on 5th November 2020) while data collection was ongoing.

### 2.4 Participants

In total, 3,817 university students completed the survey. The majority of participants were female (71%). The mean age was 23 years (± 5.87), and the majority were from a white ethnic background (85%).

### 2.5 Measures

#### 2.5.1 Generalized Anxiety Disorder-7

The Generalized Anxiety Disorder-7 (GAD-7; 20) is a 7-item scale that assesses the occurrence of anxious symptoms over the last 2 weeks. For example, “feeling nervous, anxious or on edge.” Responses are recorded on a 4-point scale (0 = *not at all* to 3 = *nearly every day*), with higher scores indicating higher levels of anxiety. The cut off points for mild, moderate, and severe levels of anxiety are scores of 5, 10, and 15, respectively. The internal consistency of the GAD-7 in this study was excellent, *α* = 0.92.

#### 2.5.2 Patient Health Questionnaire

The Patient Health Questionnaire (PHQ-9; 21) is a 9-item scale that assesses frequency of depressive symptoms over the last 2 weeks. Example items include “feeling down, depressed, or hopeless” and “thoughts that you would be better off dead.” All items are scored on a 4-point scale (0 = *not at all* to 3 = *nearly every day*), with higher scores suggesting higher levels of depressive symptoms. Cut off points of 5, 10, and 15 were used to represent mild, moderate, and moderately severe/severe depression. The internal consistency of the PHQ-9 in this study was excellent, *α* = 0.90.

#### 2.5.3 The UCLA Loneliness Scale

The UCLA loneliness scale (ULS; 22) consists of statements which measure an individual’s feelings of loneliness. Example items include: “I feel left out” and “I lack companionship.” Responses were measured on a 4-point scale (1 = *never* and 4 = *always*), with higher scores indicating higher levels of perceived loneliness. The internal consistency for this study was *α* = 0.85.

#### 2.5.4 Adapted version of the Academic Stress Scale

An adapted 5-item version of the Academic Stress Scale (ASS; 23) measures an individual’s academic stress. An example question was the extent to which the individual was stressed about “excessive workload.” Some of the original items (e.g., forgetting pencil/pen) were either dropped or adapted to reflect modern learning environments. Response options ranged from *1 = not at all stressed* to *5 = extremely stressed*, and higher scores suggest higher levels of academic stress. The internal consistency for this study was *α* = 0.78.

#### 2.5.5 The Academic Expectations Stress Inventory

Single items were taken from the Academic Expectations Stress Inventory (AESI; 24), which tapped stress related to students own expectations and those of others (e.g., teachers and parents). Example items included “I feel stressed when I am disappointed in my grades” and “I feel stressed when I know others are disappointed in my grades.” Response options ranged from *1 = never true* to *5 = always true*. The internal consistency for this study was *α* = 0.63.

#### 2.5.6 The Social Identity Scale

The Social Identity Scale (SIS; 25) consists of four items which assess the individual’s identity with their university friends. Example items include: ‘I identify with my university friends’ and ‘I am glad to be part of my university friendship group’. Response options ranged from 1 “*strongly disagree*” to 5 “*strongly agree*.” Higher scores suggest higher levels of identity. The internal consistency for this study was *α = 0.94.*

#### 2.5.7 The School Climate and School Identification Measure-Student

An adapted version of the School Climate and School Identification Measure-Student (SCASIM-St; 26) scale was used to assess university identity. The word “school” was replaced with “university.” Example items include: “I feel I belong at this university” and “I identify with this university.” Higher scores suggest higher levels of university identification. The SCASIM-St scale comprises 6 items scored on a 7-point scale (1 = “*strongly disagree*” to 7 = “*strongly agree*”). The internal consistency for this study was *α* = 0.93.

#### 2.5.8 The Exeter Identity Transition Scale

The 3-item Exeter Identity Transition Scale (EXITS; 27) measures multiple group membership. For example, “I am a member of lots of different groups at university.” Response options range from *1 = do not agree* to *7 = agree completely*. The internal consistency for this study was *α* = 0.87.

#### 2.5.9 The Intolerance of Uncertainty Scale

The 12-item Intolerance of Uncertainty Scale (IUS; 28) measures reactions to uncertain events. For example, “it frustrates me not having all the information I need.” This measure was included in 2020 dataset only. Responses options range from 1 = “*not at all a characteristic of me*” to 5 = “*entirely characteristic of me*.” The greater the overall score, the higher the intolerance of uncertainty. The internal consistency for this study was *α* = 0.91.

#### 2.5.10 Attitudes toward online teaching and learning

To ascertain students’ feelings toward online teaching and learning, a single-item was included: “How are you finding remote delivery of teaching and learning this semester?” Response options ranged from 1 “*I really dislike it*” to 5 “*I really like it*.”

#### 2.5.11 Diagnosis of COVID-19

To establish whether students have been diagnosed with COVID-19, a single item was included: “Have you had COVID-19 (coronavirus)?.” Responses were categorized as “formal diagnosis” or “no formal diagnosis.”

### 2.6 Statistical analyses

Hierarchical regression analyses were conducted to assess the contributions of academic and non-academic predictors in explaining common mental health difficulties. Hierarchical regression is robust enough to tolerate violations of normality (29). We included depression and anxiety as dependent variables. Following McIntyre et al. (6), predictor variables were entered into the model at different blocks. Each block represented a distinct cluster of associated predictors. Predictor variables were entered into the model as follows: Block 1: demographic variables (age, sex, ethnicity, higher education institution); Block 2: academic stressors (expectations and academic stress); Block 3: social identification variables (university friends identification, university identification, and multiple group membership); Block 4: social isolation; and Block 5: COVID-19 variables (intolerance of uncertainty, attitudes toward online learning, and COVID-19 diagnosis).

## 3 Results

### 3.1 Extent of mental health issues

**Figure 1:**
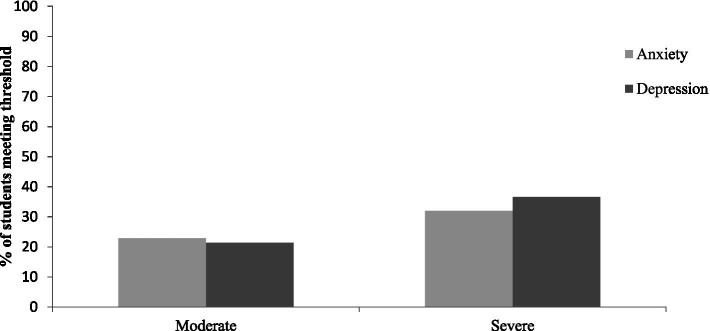
Proportion of students meeting the criteria for moderate and severe mental health symptoms.

As shown in Figure 1, using the published criteria for moderate anxiety (10–14) and depression (10–14), the proportion of students above these cut offs was 54.9% for anxiety and 58.1% for depression. Using the published criteria for severe anxiety (GAD-7: 15–21), 32.0% met the criteria for severe anxiety during the COVID-19 pandemic. Using the published criteria for moderately severe and severe depression (PHQ-9: 15–19 moderately severe and 20–27 severe), 36.6% met the criteria for moderately severe/severe depression during the COVID-19 pandemic.

### 3.2 Academic and non-academic predictors of common mental health difficulties

To understand how an accumulation of factors impact on mental health, a series of hierarchical regressions were conducted to determine which predictors were the most important determinants of symptoms. Descriptive statistics of the final sample (*n* = 3,817) are shown in Table 1.

**Table 1:** Descriptive statistics.

	Mean	Standard deviation
Academic stress	16.79	±4.54
Academic expectations	8.72	±1.59
University friends identification	13.95	±4.13
University identification	29.36	±7.82
Group membership	6.82	±4.33
Social isolation	18.99	±5.14
Intolerance of uncertainty	35.64	±10.19
Online teaching	2.56	±1.21
Anxiety	11.98	±7.31
Depression	10.95	±6.33

#### 3.2.1 Predicting symptoms of depression during the COVID-19 pandemic

The overall regression model predicted 46% of the variance in depression [*R^2^* = 0.46, *F*(13, 3,145) = 208.88, *p* < 0.001]. As reported in Table 2, at Block 1 the demographic variables explained a significant portion of variance in depression. Together, the demographic variables predicted approximately 4% of the variance in depression. Sex and ethnicity were unrelated to depression; however, age and HEI were significant predictors with higher symptoms of depression in younger students and those studying at a post-92 institution. At Block 2, academic stressors contributed significantly to the model predicting depression. Together, the academic stressors accounted for approximately 24% of the variance in depression scores. Higher levels of academic stress and expectations stress predicted higher levels of depression. At Block 3, the social identification variables contributed significantly to the model. Combined, the three social identity variables explained approximately 5% of the variance in depression scores. University identification emerged as the only significant predictor of depression symptoms. Stronger identification with university predicted lower levels of depression. Block 4 accounted for approximately 11% of the variance in depression scores. Feeling isolated was strongly associated with higher levels of depression. At Block 5, COVID-19 related variables contributed significantly to the model. Together, the three COVID-19 variables accounted for approximately 3% of the variance in depression scores. While this block added less unique variance compared to the previous blocks, it still played a role in predicting depression scores. High intolerance of uncertainty and having contracted COVID-19 were associated with higher levels of depression. Students who disliked online teaching and learning also reported higher levels of depression.

**Table 2:** Regression analysis showing the academic and non-academic predictors for depression.

Variable	Cumulative		Simultaneous	
	R^2^ change	F-change	β	*p*
Block 1				
Age	0.04	*F*(4, 3,154) = 34.47*	−0.03	0.024
Sex			0.01	0.519
Ethnicity			0.01	0.428
HEI			0.11	<0.001
Block 2				
Academic stress	0.24	*F*(2, 3,152) = 512.60*	0.22	<0.001
Academic expectations			0.06	<0.001
Block 3				
University friends identity	0.05	*F*(3, 3,149) = 69.34*	0.00	0.993
University identification			−0.07	<0.001
Multiple group membership			0.00	0.966
Block 4				
Social isolation	0.11	*F*(1, 3,148) = 620.62*	0.35	<0.001
Block 5				
Intolerance of uncertainty	0.03	*F*(3, 3,145) = 58.30*	0.17	<0.001
Diagnosis of COVID-19			−0.04	0.007
Online teaching			−0.11	<0.001

*p < 0.001.

In sum, the R^2^change for Block 2 underscores the importance of academic stressors as predictors of depression. As Block 2 has the largest R^2^change, this suggests it is the most crucial in predicting depression scores. Block 3, which included social identity variables, also contributed significantly, though to a lesser extent. The additional variance explained by Block 4 highlights the significance of social isolation. Although Block 5 explained the least amount of variance, the significant contribution of intolerance of uncertainty, contracting COVID-19, and more negative attitudes toward online learning indicates that COVID-19 related variables also affect depression scores. Thus, academic stress, academic expectations, university identification, social isolation, intolerance of uncertainty, contracting COVID-19, and more negative attitudes toward online teaching were all significant predictors of depression when controlling for all academic and non-academic stressors (see Table 2).

#### 3.2.2 Predicting symptoms of anxiety during the COVID-19 pandemic

The overall regression model predicted 49% of the variance in anxiety [*R*^2^ = 0.49, *F*(13, 3,145) = 231.71, *p* < 0.001]. As reported in Table 3, at Block 1 the demographic variables explained a significant portion of variance in anxiety. Together, the demographic variables predicted approximately 3% of the variance in anxiety. Age, sex and ethnicity were unrelated to anxiety; however, those studying at a post-92 institution reported higher levels of anxiety. At Block 2, academic stressors contributed significantly to the model predicting anxiety. Combined, the academic stressors accounted for approximately 29% of the variance in anxiety scores. Higher levels of assessment stress and expectations stress were associated with higher levels of anxiety. Although Block 3 accounted for approximately 2% of the variance, none of the social identification variables reached significance. At Block 4, social isolation contributed significantly to the model. Block 4 accounted for approximately 9% of the variance in anxiety scores. Feeling isolated was strongly associated with higher levels of anxiety. At Block 5, COVID-19 related variables contributed significantly to the model. Together, the three COVID-19 variables accounted for approximately 7% of the variance in anxiety scores. While there was no effect of having contracted COVID-19, intolerance of uncertainty was associated with higher levels of anxiety, and students who disliked online teaching and learning reported higher levels of anxiety.

**Table 3:** Regression analysis showing the academic and non-academic predictors for anxiety.

Variable	Cumulative		Simultaneous	
	R^2^ change	F-change	β	*p*
Block 1				
Age	0.03	*F*(4, 3,154) = 28.17*	0.00	0.833
Sex			0.02	0.184
Ethnicity			−0.02	0.120
HEI			0.06	<0.001
Block 2				
Academic stress	0.29	*F*(2, 3,152) = 659.29*	0.23	<0.001
Academic expectations			0.09	<0.001
Block 3				
University friends identity	0.02	*F*(3, 3,149) = 24.44*	0.02	0.125
University identification			−0.01	0.619
Multiple group membership			0.01	0.418
Block 4				
Social isolation	0.09	*F*(1, 3,148) = 469.95*	0.26	<0.001
Block 5				
Intolerance of uncertainty	0.07	*F*(3, 3,145) = 139.68*	0.31	<0.001
Diagnosis of COVID-19			−0.00	0.867
Online teaching			−0.10	<0.001

*p < 0.001.

In sum, the R^2^change for Block 2 underscores the importance of academic stressors as predictors of anxiety. As Block 2 has the largest R^2^change, this suggests it is the most crucial in predicting anxiety scores. The additional variance explained by Block 4 highlights the significance of social isolation. While Block 5 added less unique variance compared to Block 2 and Block 4, it still played a role in predicting anxiety. Thus, academic stress, academic expectations, social isolation, intolerance of uncertainty, and more negative attitudes toward online teaching were all significant predictors of anxiety when controlling for all academic and non-academic stressors (see Table 3).

## 4 Discussion

The current study aimed to investigate the mental health of university students during the COVID-19 pandemic and identify academic and non-academic predictors of common mental health difficulties. Further, we aimed to determine if factors unique to the COVID-19 pandemic (e.g., attitudes toward online teaching and COVID-19 diagnosis) predicted symptoms over and above social connectivity variables. In line with previous research [e.g., (2, 15)], there were high levels of depression and anxiety during the COVID-19 pandemic, with more than 50% experiencing levels above the clinical cut offs. In fact, nearly a third of students scored above the cut off for severe anxiety (32%), while just over a third of students scored above the cut off for moderately severe depression (36.6%). These levels of depression and anxiety are higher than those reported in our previous research using data collected via an online survey in October 2016, as we found that 20.9% met criteria for severe anxiety and 11.3% met criteria for severe depression [see (6)]. Also, according to the Adult Psychiatric Morbidity Survey in 2014, one in six people report experiencing a common mental health problem (anxiety or depression) in a given week in England (30). Thus, the incidence of common mental health problems was much higher in this student sample during the early stages of the pandemic.

Consistent with previous work [e.g., (6)], academic stress and expectations stress were associated with higher levels of depression and anxiety. Of the academic variables, academic stress was most predictive of mental distress. Our findings are consistent with previous research by Plut and colleagues who applied the JD-R model to students, finding that academic stressors contributed to low well-being and performance (7).

Although university identification was associated with lower levels of depression, identification with university friends and multiple group memberships were not associated with lower levels of depression or anxiety. These findings contrast to previous research which suggested that identification with university friends confers protection against psychological distress (6). It is possible that some students found it challenging to identify or relate to their university friends during the pandemic due to physical separation, lack of in-person interaction, and limited shared experiences, such as extracurricular activities, attending lectures and/or seminars, or socializing on campus.

Consistent with previous research [e.g., (6, 31, 32)], feelings of isolation consistently emerged as a strong predictor of poor mental health. In fact, as isolation was identified as the strongest predictor of depressive symptoms in our student sample, this aligns with the findings reported by Liu et al. (32) as social isolation had the largest effect on Australian students’ psychological wellbeing. Although social isolation was the strongest overall predictor of depression, intolerance of uncertainty was the strongest overall predictor of anxiety. Students who were intolerant of uncertainty may have found it particularly difficult to cope with the unpredictable nature of the COVID-19 pandemic, leading to heightened anxiety and depression.

Receiving a COVID-19 diagnosis was associated with higher levels of depression. Dealing with the uncertainty about recovery and the potential for experiencing on-going symptoms can be challenging and contribute to feelings of hopelessness and despair. The pivot to remote teaching and learning contributed to higher levels of common mental distress as those who disliked online teaching and learning reported higher levels of depression and anxiety. Indeed, remote learning disrupted established routines that provide stability and support for students and presented various challenges such as increased pressures to study and learn independently combined with reduced motivation levels (9). Thus, alongside the loss of usual routines of attending lectures on campus, the lack of in-person interaction with lecturers and peers may have hindered academic engagement, contributing to feelings of distress.

Our findings are consistent with the JD-R model as we found that students are subjected to a range of academic and non-academic demands that contribute to common mental health difficulties. Nevertheless, HEIs also provide resources such as opportunities to form a sense of identification with the university. Our findings suggest that enabling students to form a sense of identification their institution represents a psychological resource that improves symptoms of depression. As social isolation consistently emerged as a contributor to poor mental health, our findings suggest that fostering connections and a sense of identification within the university is important to buffer students against symptoms of common mental distress. Simply returning to campus is only a partial solution. Instead, university policy promoting connectedness is required. Wider social determinant interventions may be important in this context (33). For example, Groups 4 Education (G4E) is an evidence-based psychological intervention that directly targets mental distress that results from loneliness and social isolation (34). The implementation of such interventions in a university context would be likely to improve social wellbeing by providing students with the knowledge, skills, and confidence to increase their social connectedness. In addition to this, as remote teaching and learning contributed to higher levels of common mental distress, in any future pandemic and associated lockdowns, it would be beneficial for HEIs to cater for socially distanced in-person teaching and learning as soon as it is safe to do so. However, small changes to curricula should be implemented to increase university identification and identification with the course itself, such as embedding belonging-focussed learning activities throughout curricula and implementing more group-based projects (35). Thus, by identifying specific predictors of mental health difficulties, the study provides actionable insights related to curriculum design and student belonging interventions for universities and policymakers.

Our findings should be considered in light of several limitations. As the sample comprised students attending two universities in the North of England, generalisability is limited. Our participants may represent a specific subset of the population as they were all self-selected volunteers. The high proportion of females also suggests our sample may not be representative of the entire student population. It is also important to note that the second national lockdown in England was announced (on 31st October 2020) and implemented (on 5th November 2020) while data collection was ongoing, which may have impacted levels of distress. In addition, the proposed causal relationships should be interpreted with caution given the cross-sectional nature of the data. Last, our findings should be treated with caution due to the inclusion of abbreviated measures and single items. For example, the use of a single item for diagnosis of COVID-19 only covers the potential impact of a medical diagnosis on psychological health outcomes, rather than covering the potential influence of the fear of contagion and also the continuum of severity. Nevertheless, examining a wide range of academic and non-academic predictors meant striking a balance between including multiple predictors and examining those predictors in detail. Future research could adopt the COVID-19 Student Stress Questionnaire (developed and validated after the launch of our student survey in 2020) as this tool covers specific sources of stress featuring university students’ experiences during the pandemic, with three subscales including Relationship and Academic Life, Isolation, and Fear of Contagion (36).

To summarize, the study found high levels of self-reported symptoms of depression and anxiety among university students during the early stages of the COVID-19 pandemic. The study also comprehensively examined both academic and non-academic predictors as well as novel predictors of distress related to the COVID-19 pandemic, providing a holistic understanding of the various influences on students’ mental health. Although feelings of isolation consistently emerged as a strong predictor of poor mental health, our findings show that social-psychological pandemic-related factors other than social isolation may affect mental health, such as intolerance of uncertainty and attitudes toward online teaching. These findings therefore provide insights that could help universities and policymakers develop targeted interventions to support the mental health and well-being of university students during future crises. Following the disruption to teaching, learning and university life, together with other stressors placed on young people from the COVID-19 pandemic, there is an urgent need to support this vulnerable population both socially and academically.

## Data Availability

The original contributions presented in the study are included in the article/supplementary material, further inquiries can be directed to the corresponding author.
